# Birth Preparedness and Complication Readiness among Women with Pregnancy and Childbirth related Complications at Kenyatta National Teaching and Referral Hospital, Kenya

**DOI:** 10.24248/eahrj.v4i1.618

**Published:** 2020-06-26

**Authors:** Peter Kamwathi Ihomba, Jackim M Nyamari, Francis Ng'ang'a Murima, Tom Were

**Affiliations:** a School of Public Health, Kenyatta University, Kenya; b School of Public Health, Biomedical Sciences and Technology, Masinde Muliro University of Science and Technology; c Ministry of Health, Kenya

## Abstract

**Background:**

In developing countries, particularly those in Sub-Saharan Africa, women and newborns continue to face increased risks of mortality and morbidity during the time of pregnancy, birth and postpartum. Preparing for childbirth and being ready for complications is a key strategy in reducing maternal mortality and morbidity as this would reduce delay in obtaining skilled maternal care especially during childbirth. This survey was evaluating birth preparedness and complication readiness (BPCR) among women seeking services at Kenyatta National Teaching and Referral Hospital.

**Methods:**

A cross-sectional hospital-based study was conducted among women admitted in the antenatal and postnatal ward. Data was collected using a standardised questionnaire. A respondent was considered to have satisfactory BPCR if she reported that she had identified the place of delivery, made prior financial arrangements and organised for means of transport to place of childbirth and/or for the time of obstetric emergencies ahead of childbirth.

**Results:**

The survey recruited 353 women aged between 15 and 44 years. Majority were married (n=288, 81.6%) and unemployed (n=232, 65.7%). Additionally, most of the participants were multiparous (n=345,97.7%) and had made at least 1 visit at the Antenatal Clinic during their current pregnancy (n=331, 93.8%). The proportion of women whose BPCR was rated as satisfactory was 56.7% (95% confidence interval, (CI) 49.7% - 63.6%). Factors associated with satisfactory BPCR included: being married (OR10.66, 95%CI5.21-21.83), having post-secondary education (OR 11.52, 95% CI 6.62-20.05), being in formal employment (OR 4.14, 95%CI2.51-6.82), gestation >28 weeks (OR=1.83,95%CI1.08-3.09), multiparity (OR=1.87,95%CI1.21-2.88), visiting Ante-natal Care Clinic (OR=9.31, 95% CI 2.70-32.09)and particularly visiting the clinic more than 2 times (OR=4.43, 95% CI 2.75-7.13).

**Conclusions:**

The study documented sub-optimal BPCR. This highlights the need to review the current strategies and approaches being utilised to promote BPCR.

## BACKGROUND

Maternal mortality has continued to be a major public health problem globally. It is estimated that 830 women die daily from pregnancy and childbirth related causes. Globally, it is estimated that 303,000 maternal deaths occur annually. Sub-Saharan Africa (SSA) bears a disproportionately high burden accounting for 66% of the world's maternal deaths.^1^

Maternal deaths are thought to occur due to 3 main delays; delays in deciding to seek care, delays in reaching care, and delays in receiving care. These delays have many causes including; logistic and financial concerns, unsupportive policies and gaps in services, as well as inadequate community and family awareness and knowledge about maternal and newborn health issues.^2^ Delays in deciding to seek care may be caused by failure to recognise signs of complications, failure to perceive the severity of illness, cost considerations, previous negative experiences with the health care system, and transportation difficulties. Delays in reaching care may be created by the distance from a woman's home to a facility or provider, the condition of roads, and a lack of emergency transportation facilities. Delays in receiving care may result from unprofessional attitudes of providers, shortages of supplies and basic equipment, lack of health care personnel, and poor skills of health care providers.^3^

To address the 3 delays, implementation of Birth Preparedness and Complication Readiness (BPCR), a safe motherhood strategy has been recommended globally. It is estimated that exposure to BPCR interventions results in 18% reduction in neonatal mortality risk and a 28% reduction of in maternal mortality risk.^4^ The BPCR strategy was incorporated in the World Health Organization (WHO) antenatal care package in 2005.^5,6^ BPCR is a process of planning for birth and anticipating actions in case of obstetric emergencies in order to reduce delays in seeking skilled care.^7^ The following elements are emphasised in BPCR: deciding on the desired place of birth, preferred birth attendant, location of the closest facility for birth and in case of complications- funds for expenses related to birth and/or complications; supplies necessary for giving birth, an identified labour and birth companion; an identified support person to look after home and children while the woman is away, transport to a facility for birth or when complications arise; and identification of compatible blood donors when needed.^5,7^

Birth preparedness and complication readiness is implemented as part of focused antenatal care in Kenya. This takes the form of Emergency obstetric care (EmOC) and it includes a set of medical interventions or functions to manage life-threatening obstetric complications. At the basic level (meant to be provided in health centres), it includes intravenous (IV) administration of antibiotics, uterotonics, and anticonvulsants, manual removal of the placenta, removal of retained products of conception following miscarriage or abortion and assisted vaginal childbirth with forceps or vacuum extractor. Comprehensive emergency obstetric care, typically delivered in county hospitals includes; basic care, caesarean services, safe blood transfusion and skilled care during childbirth

Skilled care refers to the process by which a pregnant woman and her baby are provided with adequate care during pregnancy, labour, birth, and the postpartum (MacDonald and Starrs 2002). NFPA 2001b).

For this to be achieved, an enabling environment ought to be provided. Women with life threatening complications resulting from pregnancy or childbirth are prioritised and monitored closely but skilled care ought to be extended to all women during this period.

Despite such programmatic interventions, the maternal mortality in Kenya remains unacceptably high. In fact, Kenya is one of the 10 countries that accounts for nearly 60% of the global burden of maternal mortality. Currently, the Maternal Mortality Ratio (MMR) is 510 deaths per 100,000 live births. The decline in MMR over time has not been impressive considering that the MMR in 1990 was 687 deaths per 100,000 live births.^8^ The figures are a far cry from the Sustainable Development Goals (SDGs) of reducing the MMR to less than 70 per 100,000 live births between 2016 and 2030.^9^ With this background, there is urgent need to understand the BPCR practices in Kenya as well as the attendant facilitators and barriers to its implementation and uptake.

There is dearth of published literature on the status of BPCR in Kenya particularly among women attending referral hospitals. The present study aimed to fill this gap, at least in part, by documenting the current status of BPCR and factors associated with it among women seeking services at Kenyatta National Teaching and Referral Hospital (KNH).

## METHODS

### Study Design and Study Site

A hospital-based cross-sectional study was conducted between November 2014 and February 2015. The site of the study was KNH which is the largest referral hospital in East and Central Africa. The hospital has a capacity of 1,800 beds and annual inpatient coverage is estimated to be 89,000 patients. There are 6 wards that handle clients admitted for antenatal, delivery and postnatal care. The bed capacity of each of the wards is 32. Kenyatta National Hospital holds an antenatal care unit that reviews clients from Monday to Friday. Mothers undergo full antenatal profile that comprises routine investigations. Clients who are classified as high risk are monitored closely and those requiring close monitoring are admitted to an antenatal ward. Clients who come for antenatal care at the hospital have to un dergo complete antenatal profile.

### Study Population

The study population comprised of women admitted at KNH with pregnancy or childbirth related complications. Un-conscious/ semi-conscious clients and those who did not consent for this study were excluded.

### Sample Size

The hospital serves a population of over 10,000 (per year) women in their reproductive age (15-49) years (KNH, 2014).

The sample size was determined using Fisher's method as per (Mugenda and Mugenda, 2003).

n=z2pq/d2

n=desired sample size

z=standard normal deviate (1.96) that corresponds to 95% confidence level

p= the proportion in the target population with complications related to pregnancy and child birth unknown hence set at 50%.

q=1.0-p size

d=the degree of accuracy desired

n=1.962.0.50.0.50/0.052

n=384

28 questionnaires were submitted having no data inscribed.

### Data Collection

An interviewer administered to participants semi-structured questionnaires which were used to collect data on socio-demographics, obstetric attributes and antenatal care clinic's attendance. A pre-test was conducted a month before the actual study and the questionnaire translated to Kiswahili for those participants who were not conversant with English. 5 research assistants were engaged for the duration of study and were pre-trained for the study. The time taken with a given participant was variable and ranged between 15 and 25 minutes.

### Statistical Analysis

Data was entered in Microsoft Excel and analysed using STATA 13, a general purpose statistical software package created in 1985 by Stata Corp. Categorical variables were summarised using frequencies and proportions. Chi-square (χ^2^) test was used to assess the associations between the various independent variables and BPCR. The threshold for statistical significance during hypothesis testing was set at p <.05. The risk estimates are presented as Odds Ratios (OR) and the corresponding 95% Confidence Intervals (CI).

### Ethical Considerations

Ethical approval to conduct the study was obtained both from Kenyatta University Ethical Review Committee and Kenyatta National Hospital Ethical Review Board. Informed consent were sought from all the study participants after explaining to them the objectives of the study, procedures and also assuring them their right to refuse to participate in the study at any time. Participants were not required to provide their identification and filled questionnaires were stored in a lockable cabinet. Data was coded and entered safely in STATA

## RESULTS

### Socio Demographic Characteristics

A total of 353 women seeking emergency obstetric services at KNH were enrolled in the study. The age of the respondents ranged from 15 to 44 years with 23(6.5%) and 35(9.6%) of the respondents being teenagers (≤19 years) and at least 35 years. Majority of the respondents were married 288 (81.6%), 232(65.7%) unemployed and 236(67.1%) had secondary school education or lower educational qualifications

### Obstetric Characteristics

All participants in this survey were either pregnant 233(66.0%) or in the postpartum phase 120(34.0%). Of those who were pregnant, 132(56.7%) reported that their pregnancies' gestation was more than 28 weeks. Asked if they had ever experienced any obstetric complications in the past, 160(45.3%) of the participants responded on the affirmative. Investigations into the parity of the respondents revealed that majority were multiparous 345(97.7%).

### Utilisation of Antenatal Care Services

A vast majority of the women 331(93.8%) had made at least 1 visit at the antenatal clinic. The debut visits made were between 0 and 16 weeks and 17 to 28 weeks by 175(52.9%) and 117(35.3%) women respectively. 39 respondents had made their debut ANC visit in the period ranging from 29 to 40 weeks. On the number of antenatal visits made: 33(10.0%), 88(26.6%) and 73(22.1%) women had sought ANC services once, twice and thrice respectively. Moreover the proportion of women who had visited ANC 4 times were 19.6% (65) while 72 (21.8%) had made 5 visits or more. (Table 1)

**TABLE 1.** Utilisation of Antenatal Care Services

Characteristic	Frequency	%
**Did you attend antenatal care (n=353)**		
Yes	331	93.8
No	22	6.2
**Debut period in weeks (n=331)**		
0-16	175	52.9
17-28	117	35.3
29-40	39	11.8
**No. of antenatal visits (n=331)**		
1	33	10.0
2	88	26.6
3	73	22.1
4	65	19.6
≥5	72	21.8
**Received health education on danger signs (n=331)**		
Yes	263	79.5
No	68	20.5

### Birth Preparedness and Complication Readiness

Itemised assessment of BPCR showed that 259(73.4%) and 293(83.0%) of the women had, respectively, identified the venue for delivery and saved money towards delivery, prior to childbirth. Additionally, 264(74.8%) of the interviewed women reported having made arrangements for transport to the place of childbirth or for the time of obstetric emergencies ahead of childbirth. Overall, 200(56.7%) of the respondents (95%CI:49.7%-63.6%) were found to have satisfactorily prepared for childbirth and were ready for the contingent complications. (Table 2)

**TABLE 1.** Utilisation of Antenatal Care Services

BPCR Attribute	Frequency (n=353)	%
**Made a plan of where to deliver (venue of delivery)**		
Yes	259	73.4
No	94	26.6
**Saved money towards delivery**		
Yes	293	83.0
No	60	17.0
**Made arrangement for transport**		
Yes	264	74.8
No/No response	89	25.2
**Birth preparedness and complication readiness**		
Satisfactory	200	56.7
Not satisfactory	153	43.3

### Factors Associated with Birth Preparedness and Complication Readiness

#### Socio-demographic variables and birth preparedness and complication readiness

Table 3 presents the findings on the assessment of the association between BPCR and selected socio-demographic variables. A higher proportion of older women aged 30 years or more were found to be birth prepared and complications ready compared to their younger counterparts aged less than 30 years (63.5% versus 52.9% respectively). However, this association failed to attain statistical significance (Odds Ratio (OR) 1.55(95% CI 0.99-2.42), p=.054). The marital status of the respondent was statistically significantly associated with BPCR (p<.001). Married women were found to be approximately, 11 times more likely to be birth prepared and complications ready compared to the unmarried women (OR10.66,95% CI5.21-21.83). Having higher levels of education was statistically significantly associated with increased likelihood of BPCR (p<.001). A higher proportion of women who had secondary and post-secondary qualifications were prepared for birth and ready for complications when evaluated against those with lower educational qualifications (respectively, 75.1% and 20.8%, OR=11.52,95%-CI 6.62-20.05). Being in formal employment was associated with a fourfold increment in the probability of a woman being prepared for birth and ready for complications (OR=4.14,95%-CI2.51-6.82, p<.001).

**TABLE 3.** Association Between Selected Socio-demographic Factors and BPCR

Characteristic	Total	Satisfactory BPCR		OR*(95% CI†)	P-value
		Yes	No		
**Age (years)**					
≥30	126	80(63.5)	46(36.5)	1.55(0.99-2.42)	0.054
<30	227	120(52.9)	107(47.1)	Ref	
**Marital status**					
Married	288	190(66.0)	98(34.0)	10.66(5.21-21.83)	<0.001
Unmarried	65	10(15.4)	55(84.6)	Ref	
**Education**					
(Post-)Secondary	237	178(75.1)	59(24.9)	11.52(6.62-20.05)	<0.001
Primary	106	22(20.8)	84(79.2)	Ref	
**Employment**					
Formal	121	94(77.7)	27(22.3)	4.14(2.51-6.82)	<0.001
Informal	232	106(45.7)	126(54.3)	Ref	

#### Obstetric characteristics, attendance of antenatal clinic and birth preparedness and complication readiness

The study also sought to evaluate the BPCR and various obstetric attributes of the study participants. Although more women in the postpartum period were rated as birth prepared and complications ready compared to those who were pregnant at the time of the survey, this association was not statistically significant (60.0% against 54.9% respectively, OR 1.23 (95% CI 0.79-1.92), p=.336). Conversely, amongst those who were pregnant, significant variations in BPCR were observed depending on the gestation (p=.024). A woman whose gestation period was more than 28 weeks was 83% more likely to be BPCR compared to those whose gestation period was lower (OR1.83, 95%CI 1.08 -3.09). In terms of parity, having more than 1 child increased the likelihood of being BPCR by 87% (OR 1.87, 95%CI 1.21-2.88,=.004). A greater proportion of women who had experienced obstetrics complications in the past were found to prepared for birth and ready for complications compared to those who had never experienced obstetrics complications though this difference in the 2 groups was not statistically significant (60.6% versus 53.4% respectively, OR1.35(95%-CI0.88-2.06), p=.171). Attendance of antenatal clinic (ANC) resulted in a nine-fold increment in the possibility of a woman being BPCR (OR9.31,95%CI2.70-32.09, p<.001). Moreover, an increase in the number of visits was shown to significantly increase the level of BPCR. Visiting ANC more than 2 times increased the probability of BPCR by about 4 times (OR4.43,95%CI2.75-7.13, p<.001) (Table 4).

**TABLE 4.** Relationship Between Selected Obstetric Characteristics and BPCR

Characteristic	Total	Satisfactory BPCR§		OR*(95% CI†)	P-value
		Yes	No		
**Pregnant or Postpartum**					
Postpartum	120	72(60.0)	48(40.0)	1.23(0.79-1.92)	.336
Pregnant	233	128(54.9)	105(45.1)	Ref	
**Gestation (weeks)**					
>28	132	81(61.4)	51(38.6)	1.83(1.08-3.09)	.024
≤ 28	101	47(46.5)	54(53.5)	Ref	
**Parity**					
>1	182	118(64.8)	64(35.2)	1.87(1.21-2.88)	.004
0 or 1	163	81(49.7)	82(50.3)	Ref	
**Previous obstetrics complications**					
Yes	160	97(60.6)	63(39.4)	1.35(0.88-2.06)	.171
No	193	103(53.4)	90(46.6)	Ref	
**Attended antenatal care clinic**					
Yes	331	197(59.5)	134(40.5)	9.31(2.70-32.09)	<.001
No	22	3(13.6)	19(86.4)	Ref	
**No. of ANC visits**					
>2	210	152(72.4)	58(27.6)	4.43(2.75-7.13)	<.001
1 or 2	121	45(37.2)	76(62.8)	Ref	

## DISCUSSION

The current study demonstrated that over 50% of women interviewed reported to have been ready for birth and any complications that might arise during pregnancy or during labour. This proportion is slightly lower when compared to the findings of a study done in Sri Lanka where satisfactory BPCR was reported in 83.5% of the study participants.^10^ Further, a research done in East Pokot District, Midwest- Kenya documented much lower levels of BPCR: 28% of the women had satisfactory BPCR.^11^

In Banglandesh, less than a quarter (24.5%) of women were considered well prepared for birth.^12^

The disparities in the findings could be attributed to the differences in the study settings with our study being hospital based while the other studies were community-based. Moreover, the East Pokot and the Bangladesh study were conducted in hard-to-reach rural areas.^11,12^ The inconsistencies in the findings could also be attributed to the variations in the study populations. For instance, this study focused on women who were pregnant as well as those who had delivered. The study conducted in East Pokot recruited pregnant women who were on the second and third trimesters^11^ while the research conducted in Bangladesh recruited women who had delivered recently.^12^

About 3 in every 5 respondents in this study had made plans for a venue of delivery. In Adigrat, Ethiopia, 44.1% of the participants reported that they had identified a place of delivery.^13^ The proportion is much lower than what was documented in the current survey which could be a reflection of the changes that have occurred over time with regard to promoting maternal and newborn health. On the other hand, research conducted in the slum of Kolkata, West Bengal, showed that 100% of the women had identified the place of delivery. The study was community based and enrolled recently delivered women.^14^ This could probably explain the discordance in the findings.

Most of the respondents (74.8%) in this survey had made arrangements for transport to the venue of child delivery. This finding corroborates an Ethiopian study where 78.5% of the study participants had organised the mode of transportation to the venue of delivery.^15^ Just like in this study, in Kericho County, Kenya, majority of the respondents had made arrangement for transport to the health facility at birth (81.9%).^16^ On the contrary, a study done in KNH in 2008 observed that 55% of the interviewed had made prior arrangement for transport to the venue of delivery.^17^ This could be due to the government's intensification of interventions aimed at promoting health in the recent past.

In this study, about 4 in every 5 women had had made the apposite financial arrangements towards delivery and/or for obstetric emergency. Similarly, a study carried out in Ethiopia revealed that the overwhelming majority of the interviewed women (91.1%) had saved some money as part of BPCR.^15^ Noteworthy is the fact that saving money was the most prevalent BPCR practice, a finding which is consistent with the findings of studies done in other areas including Bangladesh^12^, Ethiopia^18^, Ghana^19^ and India.^20^ This probably indicates that both women and their partners clearly understand that money is required to cater for delivery expenses and also to facilitate referral in case of complications.

Being married was positively associated with satisfactory BPCR. The finding possibly highlights the positive influence of psychosocial and, perhaps, financial contributions provided by the spouses with respect to BPCR. In line with this, studies have indicated that involvement of men increases uptake of BPCR practices.^21,22^

The survey found a significant association between education and BPCR with higher qualifications positively impacting on BPCR among the participants. These finding are in concordance with those of a study conducted in India^23^ and South-eastern Nigeria.^24^ Both studies showed that mother's education level had large positive effects on BPCR. Likewise, research conducted in Tanzania found that women with primary education and above were twice as likely to be prepared for birth and complications compared to those with no formal education.^25^ Further, in Ethiopia, maternal education was found to be an independent predictors of BPCR practices with literate mothers being more likely to be prepared for birth and its complications.^26^

Being in formal employment was associated with higher levels of BPCR among the respondents. This can be closely related to education since majority of the people who are in formal employment tend to be highly educated. This might also be related to the fact that educated and/or formally employed women are more likely to have better income and negotiating power in their societies thus making their own decision in matters affecting their health.

In concordance with the results from this survey, a study done in Central Ethiopia revealed that parity increased the likelihood of BPCR being satisfactory^27^. One of the probable explanations to this is that as parity increases, the exposure to information on BPCR increases due to increased number of ANC visits among other reasons.

Also, in this study, women who attended ANC were more likely to be birth prepared. Moreover, increased visits to the ANC was a significant predictor of favourable BPCR. In concordance with the findings of this study, research conducted in the Osun State of Nigeria showed that women who had more ANC visits were more likely to be prepare for birth.^28^ Similar observations were documented in Bale Zone Ethiopia.^18^

A woman whose gestation was more than 28 weeks was more likely to have satisfactory BPCR. This, again, could be related to the increased ANC visits and subsequent exposure to information on BPCR. Other studies have also documented that ANC visits enhances BPCR.^20,24,29^ On the other hand, some studies have refuted the role of ANC in promoting BPCR. For example, a study conducted in Abia State, Nigeria, reported that high ANC attendance did not yield corresponding high BPCR knowledge and actions.^30^ This may be partly attributed to poor coverage of the content of the ANCs and possibly ineffective modes of communication between the service provider and the clients. For example, inability of the provider to convey his/her messages in the local language and the clients' not understanding well the language used by the provider. Additionally, the contents covered during ANC visits may not be comprehensive.

### 

#### Study Limitations

This survey being a hospital-based study, the findings cannot therefore be generalised to the entire community. The study site was a referral hospital and the presence of obstetric complication in the study participants may have had an influence on BPCR. As a result, the observations may not be generalisable to non-referral health facilities.

However, despite the highlighted limitations, the study provides key insights on the BPCR and associated factors among pregnant women in a referral hospital setting.

## CONCLUSIONS

The level of BPCR among women in the study population was sub-optimal. There is a need to review the current strategies and practices for promoting BPCR with a view to enhancing its up-take. The study also identified modifiable correlates of BPCR which should be put into consideration when rethinking of ways to enhance BPCR among pregnant women.
